# Home-Based Exercise Training and Cardiac Autonomic Neuropathy in Kidney Transplant Recipients with Type-II Diabetes Mellitus

**DOI:** 10.3390/life13061394

**Published:** 2023-06-14

**Authors:** Vassiliki Michou, Maria Nikodimopoulou, Vassilios Liakopoulos, Maria Anifanti, Aikaterini Papagianni, Pantelis Zembekakis, Asterios Deligiannis, Evangelia Kouidi

**Affiliations:** 1 Sports Medicine Laboratory, School of Physical Education & Sport Science, Aristotle University, 57001 Thessaloniki, Greece; vasilikimichou@yahoo.gr (V.M.); manyfant@phed.auth.gr (M.A.); adeligia@phed.auth.gr (A.D.); 2 Transplant Surgery Clinic of Aristotle University of Thessaloniki, Hippokration General Hospital, 54642 Thessaloniki, Greece; nikodimopoulou@yahoo.gr; 3 Division of Nephrology and Hypertension, 1st Department of Internal Medicine, Medical School, AHEPA Hospital, Aristotle University, 54636 Thessaloniki, Greece; liakopul@otenet.gr; 4 Department of Nephrology, Hippokration Hospital, Aristotle University, 54642 Thessaloniki, Greece; aapapagi@auth.gr; 5 Division of Internal Medicine, 1st Department of Internal Medicine, Medical School, AHEPA Hospital, Aristotle University, 54636 Thessaloniki, Greece; pzempeka@auth.gr

**Keywords:** kidney transplant recipients, cardiac autonomic neuropathy, diabetic kidney disease exercise, functional capacity

## Abstract

This randomized clinical trial aimed to examine the effects of a 6-month home-based, combined exercise training program on Cardiac Autonomic Neuropathy (CAN) in kidney transplant recipients (KTRs) with diabetes. Twenty-five KTRs (19 men (76.0%), with a mean age of 54.4 ± 11.3 years old, CAN and type II Diabetes Mellitus (DM-II)), were randomly assigned into two groups: A (n_1_ = 13 KTRs), who underwent a home-based exercise training program for 6 months, and B (n_2_ = 12 KTRs), who were assessed at the end of the study. A cardiopulmonary exercise testing (CPET), sit-to-stand test in 30 s (30-s STS), isokinetic muscle strength dynamometry, and 24-h electrocardiographic monitoring were applied to all participants, both at the baseline and at the end of the clinical trial. At first, there were no statistically significant differences between groups. After 6 months, group A showed higher values in exercise time by 8.7% (*p* = 0.02), VO_2_peak by 7.3% (*p* < 0.05), 30-s STS by 12.0% (*p* < 0.05), upper limb strength by 46.1% (*p* < 0.05), and lower limb strength by 24.6% (*p* = 0.02), respectively, compared to the B group. Furthermore, inter-group changes at the end of the 6-month study indicated that group A statistically increased the standard deviation of R-R intervals (SDNN) by 30.3% (*p* = 0.01), root mean square of successive differences between normal heartbeats (rMSSD) by 32.0% (*p* = 0.03), number of pairs of successive NN (R-R) intervals that differ by more than 50 ms (pNN50) by 29.0% (*p* = 0.04), high frequency (HF (ms^2^)) by 21.6% (*p* < 0.05), HF (n.u.) by 48.5% (*p* = 0.01), and turbulence slope (TS) by 22.5% (*p* = 0.02), and decreased the low frequency (LF (ms^2^)) by 13.2% (*p* = 0.01), LF (n.u.) by 24.9% (*p* = 0.04), and LF/HF ratio by 24% (*p* = 0.01), compared to group B. Linear regression analysis after the 6-month study showed that there was a strong positive correlation between VO_2_peak and SDNN (r = 0.701, *p* < 0.05) in group A. Moreover, multiple regression analysis showed that KTRs’ participation in the exercise program showed favorable modifications to sympathovagal balance and aerobic capacity, as measured with SDNN and VO2peak, respectively. To summarize, diabetic KTRs’ cardiac autonomic function and functional capacity can be improved after a home-based long-term exercise training program.

## 1. Introduction

Kidney Transplantation (KT) is a more effective therapeutic option for patients with end-stage kidney disease (ESKD) (Glomerular Filtration Rate (GFR) < 15 mL/min/1.73 m^2^) [1] when compared to dialysis in terms of functional capacity and physical activity status [2], quality of life [3], sympathovagal activity [4], and survival rates [5]. Cardiac Autonomic Neuropathy (CAN) is a severe complication of Diabetes Mellitus (DM) that is also common in the Chronic Kidney Disease (CKD) population. It is well known that patients with CAN are at high risk for arrhythmias, cardiovascular morbidity, and mortality [6,7]. According to previous studies, KT induces favorable alterations in sympathetic and vagal nerve activity and sympathovagal balance [8,9,10]. However, KT recipients (KTRs), primarily due to the use of maintenance immunosuppression [11,12] are dealing with many non- or modifiable cardiovascular risk factors after kidney transplantation, such as impaired glucose tolerance, dyslipidemia and hypertension [12,13].

In addition, lower levels of physical activity and poor fitness are integral characteristics of KTRs, which also deteriorates their quality of life [14]. In the KTR population, reduced physical activity has been strongly associated with increased cardiovascular risk and all-cause mortality. Conversely, higher physical activity levels were found to be positively correlated with increased functional capacity and exercise tolerance [11], optimal kidney graft function, especially during the first year after transplantation [15], and improved cardiac autonomic regulation [16]. Although KTRs can modestly improve their fitness after transplantation compared to patients with advanced CKD, relatively few maintain these fitness activity levels [2]. This is primarily due to various environmental and individual factors, such as fear of impending graft failure, comorbidities, and immunosuppressive drugs (particularly corticosteroids).

Even though the effects of long-term exercise training programs have been thoroughly investigated in DM or CKD patients, there is a lack of evidence regarding the impact of systemic exercise training on cardiac autonomic dysfunction in KTRs with DM. Hence, the primary aim of the study was to examine the effects of a long-term, home-based, combined exercise training program on CAN in diabetic KTRs. A secondary aim was to evaluate the exercise training-induced adaptations in functional capacity and physical fitness parameters and to explore their impact on CAN indices in these patients.

## 2. Materials and Methods

### 2.1. Patients

KTRs were recruited from the Transplant Surgery Clinic of the Hippokration General Hospital, Thessaloniki, Greece, and were screened for eligibility. KT patients’ inclusion criteria were as follows: Age > 18 years old, diagnosed with CAN and DM-2 vintage over at least 6 months. KTRs with age > 70 years, Body Mass Index (BMI) over 40 kg/m^2^, unstable hypertension (acceptable upper limits of blood pressure (BP) values were 140/90 mmHg without a sudden BP fall or rise that can cause hemodynamic instability) and/or angina (diagnosed based on the medical history and an exercise ECG), musculoskeletal problems that may limit participants’ ability to exercise, severe anemia (Hematocrit below 25%), lack of obedience with medications, receiving medication that affects the Autonomic Nervous System (ANS), and previous participation in a similar long-term exercise program were excluded.

### 2.2. Sample Size Estimation

The required sample size was estimated based on a similar study by Kouidi et al. [16] in the KT patient population. We hypothesized that there were significant differences between groups A and B in 24 h-Holter monitoring and functional capacity indices. By using the two-tailed test of significance (with the significance level at *p* < 0.05), to achieve a power of 80%, we found that a total of 12 individuals per group were required. According to the sample size estimation, the goal was to recruit 30 patients, 15 patients into each group, assuming a 20% dropout rate. In our study, we recruited and evaluated a total of 25 patients that were randomly assigned into two non-equal groups (A group = 13 individuals, B group = 12 individuals). Post-hoc power analysis, by using a two-tailed test of significance, showed that the trial had a power of 70.6–73.1% for functional capacity evaluation (according to VO_2_peak and 30-s STS), dynamometry, and HRV-HRT evaluation, and the required post-hoc sample was found to be 24 patients (12 in each group) at the end of the 6-month home-based exercise training.

### 2.3. Study Design

Initially, KTRs who met the above inclusion criteria underwent a clinical examination (including a review of their medical history and anthropometric (weight and height) measurements) recording of a 12-lead electrocardiogram (ECG)), sit-to-stand test in 30 s (30-s STS) to assess physical function, cardiopulmonary exercise testing (CPET) for the evaluation of the maximum oxygen uptake (VO_2_peak), dynamometry to determine the muscle strength of both upper and lower limbs, and a 24-h ambulatory ECG monitoring for heart rate variability (HRV) and heart rate turbulence (HRT) analysis at the Sports Medicine Laboratory of the Department of Physical Education and Sports Science of the Aristotle University of Thessaloniki, Greece [17]. After baseline measurements, which took place in October 2019, patients were randomly assigned (on 28 October 2019) to either an exercise (A group) or a control group (B group). For the randomization process, the www.randomizer.org website was used. KTRs in the A group underwent a 6-month mixed-type exercise training program at home with a supervised, progressively increasing workload. In contrast, the B group continued to receive the usual care and avoided participation in any structured exercise program.

In the follow-up period, all baseline assessments were repeated. The same researcher conducted all the above measurements, blinded to group allocation. This randomized controlled trial protocol was approved by the Ethics Committee of the Aristotle University of Thessaloniki (Protocol number: 117461/2019). Before enrollment, all participants received all necessary study information and provided written informed consent. In addition, KTRs’ medication was asked to remain stable throughout the study. The clinical trial started in October 2019 and ended in May 2020.

### 2.4. Body Weight and Height Measurements

Initially and after 6 months, the weight (kg) and height (cm) of the diabetic KTRs were measured using the SECA electronic scale.

### 2.5. Sit-to-Stand Test in 30 s

The 30-s STS is a widely used, valid, and reliable measure of lower extremity strength. At the same time, it is considered an individual’s physical capacity predictor for various pathological conditions. Older subjects can perform the 30-s STS in the same pattern, regardless of execution speed, compared to younger subjects [18]. KTRs were instructed to sit on a steel chair with a 45 cm height, non-adjusted for each participant [19], with their feet touching the floor, their knee joint at 90 degrees, their back touching the chair’s back, and their arms crossed on the shoulders at chest height. On the “go” command, KTRs were asked to execute as many repetitions as possible of sitting down and getting up from a chair within the 30-s time frame. The more repetitions the participant performed, the higher the score was. Data were collected as the number of repetitions within 30 s.

This instrument has moderate to high test-retest reliability (intraclass correlation coefficient (ICC) = 0.84–0.92) and moderate construct validity (r = 0.78) for older adults [20], while according to Lein [21], 30-s STS has concurrent, convergent, and discriminate validity for younger adults.

### 2.6. Isokinetic Strength of Limbs

The Concept2 DYNO isokinetic dynamometer (Concept2, Morrisville, VT, USA) was used to assess upper and lower limb muscle strength. Patients from a seating position on the dynamometer bench, after first adjusting the height of the seat, the handle, and the resistance, first performed three repetitions for the upper limbs and three repetitions for the lower limbs (mild intensity) to familiarize themselves with the test. They then performed 3 maximal pull–push efforts for the upper limbs and 3 maximal pull–push efforts for the lower limbs. The average of 3 upper and lower extremity efforts was recorded in watts.

### 2.7. Cardiorespiratory Exercise Testing

KTRs’ functional capacity was assessed using a treadmill CPET based on the Bruce protocol [22]. The CPET occurred during morning hours. The ECG (GE Medical Systems, Milwaukee, WI, USA) was continuously monitored through the CPET trial. At the end of each 3-min stage, according to the Bruce Protocol, blood pressure was also measured. Furthermore, the Med Graphics Breeze Suite CPX Ultima (Medical Graphics Corp, Milwaukee, WI, USA) was used to measure the breath-by-breath gas exchange. The gas indicators that were examined were the peak oxygen uptake (VO_2_peak), pulmonary ventilation (VE), ventilatory equivalents for oxygen (VE/VO_2_) and carbon dioxide (VE/VCO_2_), and the ratio between VO_2_ and maximum HR (VO_2_/HRmax). The endpoint was set as the respiratory exchange ratio ≥ 1.10 and the oxygen plateau during maximal exercise.

### 2.8. 24-h Holter Monitoring

KTRs underwent a 24 h ambulatory ECG recording to estimate the cardiac autonomic function using a small portable 3-channel (with seven electrodes) ECG Holter device (SEER 1000, GE Healthcare, Chalfont St Giles, UK). For the accuracy of the 24-h Holter analysis, all KTRs were asked to avoid consuming alcohol for at least 12 h and to refrain from vigorous physical activity for at least 24 h before the scheduled appointment in the Sports Medicine Lab, Greece. Data were stored and analyzed using the Cardio Day software (GE Healthcare, Chalfont St Giles, UK) to estimate Heart Rate Variability (HRV) indices in the Time and Frequency Domain and Heart Rate Turbulence (HRT). Regarding HRV time domain analysis, four variables were evaluated [23]:

- The standard deviation of R-R (the time intervals between two successive heartbeats) intervals (SDNN).
- The standard deviation of R-R intervals calculated every 5 min (SDANN).
- The square root of the mean sum of the squares of the differences between consecutive intervals R-R (rMSSD).
- The percentage of successive RR intervals higher than 50 ms (pNN50).

Similarly, from the HRV frequency domain analysis, the following five indices were estimated [23]:

- The total frequency power (TP).
- The very low-frequency power (VLF) (<0.003–0.04 Hz).
- The low-frequency power (LF) (0.04–0.15 Hz).
- The high-frequency power (HF) (0.15–0.4 Hz).
- The frequency ratio (LF/HF)

SDNN, SDANN, and LF/HF are indices that describe the sympathetic/parasympathetic balance of cardiac ANS; HF, rMSSD, and pNN50 are indices that represent the vagal nerve activity, while LF and VLF are indices that describe sympathetic nerve activity (23). However, according to some authors, LF is also modulated by both sympathetic and parasympathetic nerve activity [24,25,26]. This may be explained by the Sympathetic Nervous System (SNS) not producing frequency rates above 0.1 Hz. The 0.1–0.15 Hz frequency range indicates parasympathetic influence in this range and a negative correlation with heart rate [23].

In addition, the HRT indicators analyzed were the turbulence onset (TO) index and the turbulence slope (TS) index [17,27,28]. The TO index shows how much the heart’s atria speed up after a ventricular premature complex (VPC) and the percent relative change in the mean of the two R-R intervals before and after a ventricular premature beat (VPB). TS index represents the rate of deceleration of the sinus sequences, followed by the acceleration of the sinuses. It is the slope of the steepest regression line and is calculated based on the sequence of every 5 consecutive R-R intervals after a VPB within 15 R-R intervals after the VPB [27,29,30].

### 2.9. Exercise Training Program

**Figure 1:**
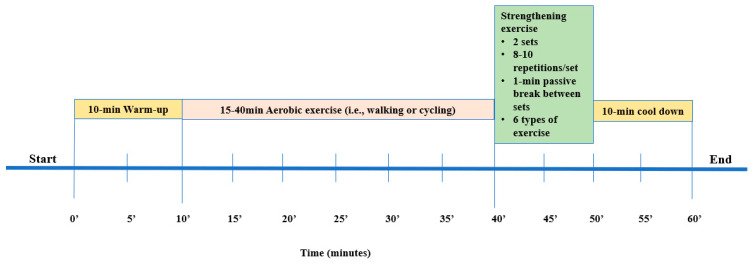
The exercise program.

According to World Health Organization, KTRs should perform at least 150 min of moderate or 75 min of high-intensity exercise or an equivalent combination of moderate and high-intensity aerobic activity per week [31]. Several studies suggest an aerobic exercise training protocol for 20 to 60 min, 2–3 days a week, on non-consecutive days, for 1 to 12 months, with an intensity equal to 50–80% of VO2peak or 40–80% of HRmax [11,32]. In this study, Group A followed a 6-month home-based exercise training program three times per week (Figure 1). The exercise program consisted of 3 combined (aerobic and strengthening) exercise sessions per week. Each exercise session was 60–70 min long, starting with a 10-min warm-up and ending with 10-min cool down exercises (upper and lower extremity stretches). The intensity was moderate, at 50–70% of the predicted VO_2_peak achieved during CPET. Moreover, during the first week, all KTRs had three familiarization sessions with accredited physical education trainers with expertise in exercise rehabilitation, and then they received a detailed information exercise booklet.

Cycling on a stationary bicycle or walking was suggested as aerobic exercise. KTRs were instructed to start exercising progressively from 15 to 40 min before the end of the 6-month exercise program. The goal was to gradually increase the exercise time by 5 min every two weeks [17]. A total of six dynamic muscle-strengthening exercises (2 sets, 8–10 repetitions per session) were included in the strength exercise part. More precisely, with a progressive sequence, KTRs started strengthening exercises from sitting to standing position (divided into 3 phases, based on a similar study protocol [17]), and after the initially gained adaptations, they exercised by using balls, bands, and dumbbells.

To ensure KTRs’ autonomy, this exercise training program was individualized. The researcher monitored the program adherence and progress either weekly via phone or video call or monthly via a home visit (wherever possible) to record improvements and provide modifications to the exercise program. Individual diaries were also given to KTRs, to better describe the total frequency, duration, and type of each exercise session. In case of a missed exercise session for medical or other reasons, KTRs were also instructed to give written reports in their diaries. In addition, similar to a previous home-based exercise protocol (17). At the beginning of each exercise session, KTRs were instructed to measure HR, blood pressure, and glucose levels and note their measurements in their diaries. However, for safety reasons, in case of unstable blood glucose levels (< 70 mg/dL or >130 mg/dL), sudden illness, dizziness, or breathlessness, KTRs were advised to skip the current exercise session.

### 2.10. 5-Point Likert Scale

To assess the A group’s subjective satisfaction after the end of the exercise program, a 5-point Likert scale was applied. KTRs were asked to show their level of satisfaction (from very satisfied to very unsatisfied) with the given statement on the 5-metric scale [33].

### 2.11. Statistical Analysis

To assess the variables’ normal distribution, the Kolmogorov–Smirnov test was applied. A two-way ANOVA with repeated measures was applied to extract mean differences within time and between A and B groups. A *t*-test for independent samples was also used to examine differences between groups A and B. Intra- and inter-observer variability was defined by the intraclass correlation coefficient (CCI) and 95% confidence intervals (Cl). The positive or negative correlation between variables that statistically improved over time was evaluated by linear regression analysis. Multiple linear regression analysis was used to determine the impact of confounding factors on results. Data were expressed as the mean ± standard deviation for normally distributed variables. The significance level for accepting or not having a statistically significant difference for all statistical tests was set at *p* < 0.05. Statistical analysis was performed using the IBM Statistical Package for Social Sciences (IBM Corp. Released 2020. IBM SPSS Statistics for Windows, Version 27.0. Armonk, NY, USA).

## 3. Results

### 3.1. KTRs’ Characteristics

**Figure 2:**
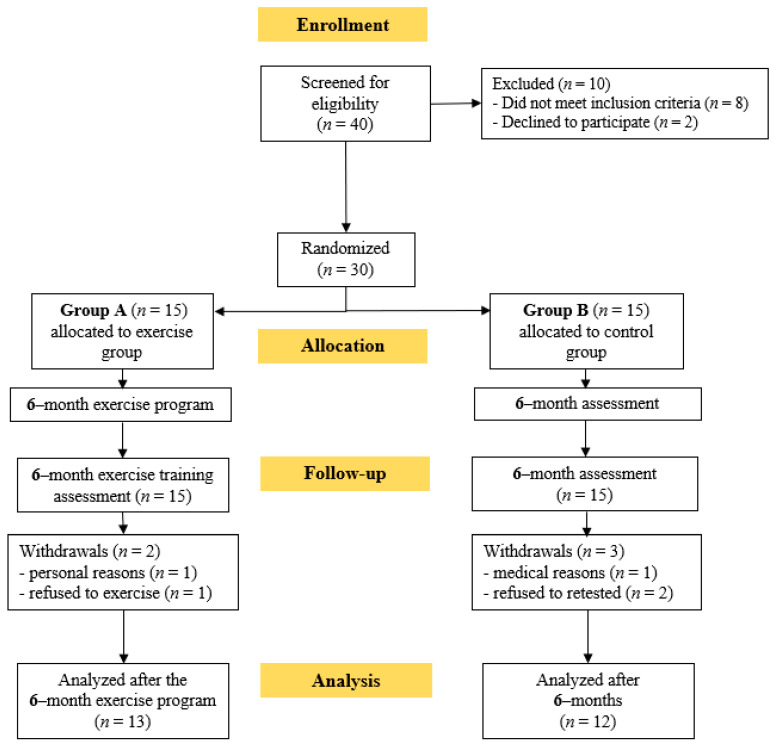
Flow chart consort diagram of the study.

At first, 40 KTRs were screened for eligibility. Thirty of these KTRs who met the inclusion criteria and volunteered to participate in this study were randomly assigned to either group A or group B. Two patients from group A and three from group B withdrew through the follow-up period due to personal reasons and refusion to exercise or be retested; therefore 25 KTRs completed the study (Figure 2). During the 6-month home-based exercise protocol, no exercise-induced musculoskeletal or cardiovascular complications were noticed. KTRs’ baseline characteristics are shown in Table 1.

### 3.2. Cardiorespiratory Efficiency

After the 6-month home-based exercise program, group A showed a significant increase in exercise time by 8.6% (*p* < 0.05), METs by 6.3% (*p* = 0.03), VO_2_peak by 7.8% (*p* = 0.01), and exercise HR by 6.4% (*p* < 0.05), while lower values were noticed in resting HR by 3.7% (*p* < 0.05) and exercise SBP by 4.8% (*p* < 0.05). At baseline, there was no statistically significant difference in any cardiorespiratory efficiency indicator between groups A and B. In contrast, inter-group changes at the end of the study showed that group A statistically increased exercise time by 8.7% (*p* = 0.02), METs by 6.6% (*p* = 0.04), VO_2_peak by 7.3% (*p* < 0.05), and exercise HR by 6.9% (*p* < 0.05), and decreased resting SBP by 3.8% (*p* = 0.01) and SBPmax by 4.5% (*p* = 0.03), compared to group B (Table 2).

### 3.3. Dynamometry and Functional Capacity

At baseline, there were no statistically significant differences in functional capacity (as measured by 30-s STS) and isokinetic dynamometry measurements between groups A and B. After a 6-month exercise program, group A showed a significant increase in 30-s STS by 10.2% (*p* = 0.01), upper limb strength by 43.5% (*p* < 0.05), and lower limb strength by 21.1% (*p* = 0.01). Regarding the inter-group changes between A and B groups at the end of the study, results for the A group showed a favorable increase in 30-s STS by 12.0% (*p* < 0.05), upper limb strength by 46.1% (*p* < 0.05), and lower limb strength by 24.6% (*p* = 0.02), compared to the B group (Table 3).

### 3.4. 24-h HRV and HRT Analysis

Group A results from the HRV and HRT analysis showed significant improvements in TP (increased by 5.8%, *p* = 0.01), SDNN (increased by 29.5%, *p* < 0.05), SDANN (increased by 31.8%, *p* = 0.01), rMSSD (increased by 34.1%, *p* = 0.03), VLF (decreased by 26.5%, *p* = 0.01), LF (ms^2^ (decreased by 13.2%, *p* < 0.05)), HF (ms^2^ (increased by 20.6%, *p* = 0.02)), LF (n.u. (decreased by 28.4%, *p* = 0.02)), HF (n.u. (increased by 49.0%, *p* < 0.05)), LF/HF ratio (decreased by 20.8%, *p* < 0.05), and TS (increased by 17.4%, *p* < 0.05). In contrast, there was no statistically significant difference in any HRV or HRT index in the B group after the end of the study (Table 4). Furthermore, inter-group changes at the end of the 6-month home-based exercise program indicated that group A statistically increased TP by 9.9% (*p* < 0.05), SDNN by 30.3% (*p* = 0.01), SDANN by 32.9% (*p* < 0.05), rMSSD by 32.0% (*p* = 0.03), pNN50 by 29.0% (*p* = 0.04), HF (ms^2^) by 21.6% (*p* < 0.05), HF (n.u.) by 48.5% (*p* = 0.01), and TS by 22.5% (*p* = 0.02), and decreased in VLF by 26.2% (*p* = 0.03), LF (ms2) by 13.2% (*p* = 0.01), LF (n.u.) by 24.9% (*p* = 0.04), and LF/HF ratio by 24% (*p* = 0.01), compared to the B group.

### 3.5. Linear Regression Analysis

**Figure 3:**
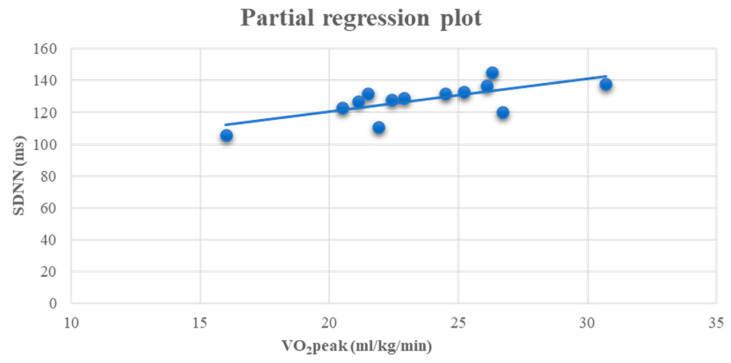
Linear regression analysis between the VO_2_peak (ml/kg/min) and SDNN (ms) (r = 0.701, *p* < 0.05) after 6 months in the A group.

At the end of the study, a positive linear relationship was found in group A between SDNN and VO_2_peak (r = 0.701, *p* < 0.05) (Figure 3).

### 3.6. Multiple Linear Regression Analysis

Lastly, at the end of the study, multiple linear regression analysis was performed to explore the relationship between sympathovagal balance, as measured with SDNN, and aerobic capacity, as measured with VO_2_peak, with a variety of independent variables in the A group. By using SDNN as the dependent variable, the results showed that higher values of VO_2_peak (*p* < 0.05) and lower limb strength (*p* = 0.02) had a statistically significant contribution to the model (Table 5). More precisely, results revealed that 81.7% of the variability observed in SDNN was explained by the regression model (R^2^ = 0.817, F = 9.245, *p* <0.05). Moreover, by using VO_2_peak, as a subordinate variable, the analysis showed that higher values of SDNN (*p* = 0.01) had a statistically significant contribution to the model (Table 6), which explained 90.4% of the total variance (R^2^ = 0.904, F = 15.430, *p* < 0.05).

### 3.7. Likert Scale Analysis

KTRs in the A group stated that they felt very satisfied after the end of the 6-month home-based exercise program.

## 4. Discussion

This randomized clinical trial aimed to investigate the effects of a 6-month home-based exercise program on CAN and functional capacity of KTRs with DM-II. The major finding of the present study is that a long-term exercise program has favorable improvements in sympathovagal balance and vagal and sympathetic nerve activity in diabetic KTRs.

In general, ANS plays a vital role in the cardiovascular response to acute exercise. The central command and the baroreceptor reflex regulating blood pressure during exercise are both essential in determining the cardiovascular response to match the accuracy of systemic oxygen supply with metabolic requirements [34]. More precisely, heart rate and cardiac output increase during exercise to increase blood pressure. As a result, the cardiovascular system leads to blood distribution, so that most of it goes to skeletal muscles. As the duration and intensity of exercise increase, vasoconstriction in splanchnic organs and kidneys is also observed [35,36]. When the exercise intensity approaches approximately 75% of VO2max, increased sympathetic activation leads to the release of arginine vasopressin (which contributes to water reabsorption and blood pressure increase) and kidney blood flow reduction in proportion to exercise intensity [37,38].

Furthermore, during acute exercise (as a form of stress), cardiac sympathetic activation and parasympathetic withdrawal lead to HR acceleration, while as the exercise intensity changes to moderate (50–60% of the VO2max), the cardiac vagal activity significantly decreases [39]. After exercise, there is a phase of immediate recovery, where cardiac sympathetic activity decreases and vagal tone increases. Fast vagal reactivation after exercise cessation depends on the exercise intensity and duration. HRV recovers in a few minutes if the exercise’s intensity is low, while HRV recovery may last even two days if the intensity is high [40,41,42]. In contrast, systematic or chronic exercise is strongly correlated with better physical fitness, higher vagal dominance, and improved HRV.

Even though the effects of short- and long-term exercise on cardiac ANS mechanisms are not yet fully elucidated [43], research has shown that a series of neurotransmitters (such as catecholamines) and inhibitors, i.e., nitric oxide (NO) [44] and angiotensin II [45], can affect cardiac sympathovagal balance. During exercise, NO bioavailability and the concentration of catecholamines [46,47] are highly increased due to the activation of the Sympathetic Nervous System and the activation of pulmonary baroreceptors, with a consequent further increase in heart rate and stroke volume. In contrast, angiotensin II secretion is suppressed, and thus it is considered a cardiac vagal tone inhibitor [45]. According to the above references, systemic exercise training can increase vagal tone, with a subsequent decrease in sympathetic nerve activity. These results indicate that long-term exercise can modulate cardiac ANS function, both in healthy [48] and diseased populations [49,50]. However, even though long-term exercise is recommended for patients with DM and CAN, there are limited clinical trials to evaluate the effects of exercise in cardiac autonomic control [43], while the exact mechanisms involved in the phenomenal plasticity of the cardiac ANS remain unidentified.

Our study is the first study that evaluated the results of long-term exercise training in diabetic KTRs with CAN. Our results showed that a 6-month home-based exercise program improved indices reflecting sympathovagal activity, leading to a consequent sympathovagal balance after training. According to the existing literature, exercise has been previously applied in the KTR population, with the goal of reducing cardiovascular risk after transplantation and optimal cardiac autonomic function. Kouidi et al. [16] showed that a 6-month exercise training can significantly improve indices of HRV (i.e., SDNN) and the Baroreflex Sensitivity Test (BST) and VO_2_peak in KTRs. Meanwhile, in the study of Barroso et al. [51], a mixed-type exercise program led to significant improvements in the LF and LF/HF ratio, but not in SDNN. SDNN is considered ‘’ a gold-standard’’ variable of HRV evaluation and its reduction has been strongly correlated with high cardiovascular morbidity and mortality [52,53]. In the study of Moraes Dias et al. [54], KTRs in the exercise group had significantly higher values in HRV time (as demonstrated by SDNN and rMSSD) and frequency (as represented by HF and LF) domain indices. Despite the emerging benefits of KTRs’ cardiac autonomic modulation, these studies did not include patients with DM, nor did they implement a home exercise protocol. In our study, a significant increase in HRV indices was observed in the exercise group both in time and frequency domain indices. Therefore, the participation of KTRs with DM-II in an appropriately designed home-based exercise program can increase cardiac ANS activity and improve CAN.

Furthermore, our study is the first that evaluates HRT and the effects of a long-term exercise program in diabetic KTRs. More precisely, our study revealed a significant increase in the TS index of HRT in the exercising KTRs. HRT is a biphasic reaction of ventricular premature contraction [30], which has significant prognostic value in chronic heart failure (CHF), especially when it is combined with HRV and Heart Rate Recovery [55,56]. HRT, by using the TS and TO indexes, represents vagal nerve activity, while it is considered a total mortality-independent factor [57]. According to Watanabe et al. [27], HRT (as represented by TO and TS variables) strongly correlates with HRV indices such as SDNN and rMSSD. Piotrowicz et al. [56] were among the first to evaluate the impact of exercise on HRT and the first to analyze the effects of an 8-week exercise training on HRV, HRT, and HRR simultaneously in patients with CHF. HRT has been also assessed in patients with DM and idiopathic dilated cardiomyopathy [27]. In a previous study of diabetic CAN, Lin et al. [58] estimated and compared the Ewing test, HRV, and HRT indices to diagnose CAN in 90 DM patients. Results of this study showed that by using both HRV and HRT analysis, CAN was diagnosed in 56.6% and 52.2% of DM patients, respectively. Based on these results, diagnostic sensitivity for CAN can be increased up to 98.0% when combined with TS and SDNN indexes. Similarly, in the study of Balcioglu et al. [59] decreased TS, but no TO, was detected in DM-II patients with and without CAN. This discrepancy might be explained by the fact that sympathetic nerve activity regulates TO or TO may not be influenced by CAN. This might possibly explain the unchanged TO index in our study.

In addition, our study revealed statistically significant inter- and intra-group improvements in cardiorespiratory efficacy, muscle strength, and physical function. Few studies have previously investigated the effects of exercise on these variables in the KTR population. Painter et al. [60,61] showed that an 11-month home-based exercise program, in a total of 167 KTRs, with an approximately 1-month KT vintage, significantly improved VO_2_max, muscle strength, and quality of life. Karelis et al. [62] investigated the effects and feasibility of resistance exercise training in KTRs and found favorable improvements in VO_2_max and upper and lower muscle strength, while it did not decrease cardiometabolic risk. Riess et al. [63] also revealed that a 12-week endurance training statistically improved exercise tolerance and muscle strength, while Lima et al. [64], implementing a mixed-type exercise program for 12 weeks, also observed an increase in aerobic capacity, limb muscle strength, body composition, and renal function. They even pointed out that combining aerobic training with resistance exercise may protect against the onset or progression of cardiovascular disease, graft dysfunction, and metabolic complications following transplantation.

Moreover, according to multiple linear regression analysis, our study revealed significant correlations between SDNN and VO_2_peak and SDNN and lower limb strength in the A group in the follow-up period. These results indicate that improvements in cardiorespiratory efficiency and muscle strength affect cardiac sympathovagal balance. Similarly, Kouidi et al. [16] noticed a positive correlation between SDNN and VO_2_peak after the end of the exercise program. According to Picard et al. [65], endurance training demonstrates strong benefits on HRV indices, such as SDNN. Meanwhile, improvements in VO_2_peak, HbA1c, and BP are also linked to HRV modulation after exercise in DM-II patients [65].

Indeed, mixed-type exercise appears feasible and clinically beneficial in this patient population. Even though de Smet and Van Craenenbroeck [11] reported that evidence for the long-term effects of exercise in KTRs is limited, recent systematic reviews by Oguchi et al. [66] and Calella et al. [32] conclude that systematic long-term exercise training demonstrates favorable effects on KTRs’ aerobic and functional capacity. Greenwood et al. [67], in the ExeRT pilot study, noticed that a 3-month mixed-type exercise program statistically improved VO_2_peak in the aerobic exercise group and VO_2_peak, 60-s STS, and isometric muscle strength in the resistance exercise group compared to the controls. In contrast, in a relatively recent randomized clinical trial, O’Connor et al. [68] demonstrated an improvement in aerobic capacity following an unsupervised moderate-intensity aerobic exercise program lasting 12 weeks. During the follow-up period, there were statistically significant differences in VO_2_peak values between the exercise and control groups.

To summarize, our study has both strengths and limitations. This study is the first study that evaluates the effects of a long-term exercise program on the cardiac ANS activity of diabetic KTRs. A previous similar study on diabetic hemodialysis patients with CAN showed favorable effects on the sympathovagal activity of the heart and aerobic capacity [17]. These results, combined with the highly increased global prevalence of CKD and DM, highlight the importance of early diagnosis of CAN and the application of alternative therapeutic methods. In contrast, the limitations of the study are the small sample size, primarily due to the difficulties in recruiting diabetic KTRs for a long-term home-based exercise program. Larger randomized controlled trials are of great importance, primarily since higher levels of KTRs’ physical activity are significantly correlated with improved KTR outcomes [69]. Lastly, our study did not evaluate the prognostic value of long-term exercise.

## 5. Conclusions

In conclusion, a 6-month home-based mixed-type exercise training program can improve cardiac autonomic function and functional capacity in diabetic KTRs. Physical inactivity, poor physical functioning, and increased cardiovascular risk are significant targets in the attempt to improve HRV and clinical outcomes after KT [11]. KTRs should regularly exercise, as sedentary behavior is a common contributor to cardiovascular disease. Moreover, considering that long-term immunosuppressive therapy is associated with many complications, including weight loss, osteoporosis, and sarcopenia, regular physical activity can provide significant health benefits. However, future studies are needed to better define the association between exercise training and improved cardiac ANS function, lower cardiovascular risk, and mortality in KTRs.

## Figures and Tables

**Table 1:** KTRs’ baseline demographic and clinical data.

	Group A (n_A_ = 13)			Group B (n_B_ = 12)			A vs. B Group	
	Baseline	Follow-Up	*p*-Value	Baseline	Follow-Up	*p*-Value	Pre	Post
Clinical Characteristics								
Sex (male/female)	10/3	-		9/3	-		*p* = 0.66	
Age (years)	54.9 ± 9.9	-		54.0 ± 12.7	-		*p* = 0.91	
Height (cm)	1.6 ± 0.8	-		1.6 ± 0.5	-		*p* = 0.42	
Weight (Kg)	72.9 ± 11.5	70.7 ± 11.4	*p* = 0.88	73.6 ± 9.1	73.2 ± 6.9	*p* = 0.55	*p* = 0.87	*p* = 0.66
BMI (Kg/m^2^)	24.8 ± 3.9	24.6 ± 2.4	*p* = 0.74	25.6 ± 2.0	25.7 ± 2.1	*p* = 0.83	*p* = 0.35	*p* = 0.12
eGFR-CKD-EPI equation (ml/min)	61.4 ± 8.6	60.8 ± 3.9	*p* = 0.22	60.8 ± 9.1	60.0 ± 4.3	*p* = 0.78	*p* = 0.23	*p* = 0.48
Stage of diabetic nephropathy -Stage 3 -Stage 4	84.6% (11/13) 15.4% (2/13)	- -		66.6% (8/12) 16.6% (2/12)	- -		*p* = 0.77 *p* = 0.64	
KT vintage (months)	49.1 ± 19.1	-		48.8 ± 20.3	-		*p* = 0.68	
HD vintage (months)	77.8 ± 39.1	-		76.9 ± 45.7	-		*p* = 0.24	
Primary causes of ESKD - Diabetes mellitus - Hypertension - Polycystic Kidney Disease - Glomerulonephritis - Nephrosclerosis - Reflux nephropathy - others	61.5% (8/13) 38.4% (5/13) 23.0% (3/13) 15.3% (2/13) 7.6% (1/13) 0.0% (0/13) 7.6% (1/13)	- - - - - - -		58.3% (7/12) 33.3% (4/12) 25.0% (3/12) 16.6% (2/12) 0.0% (0/12) 16.6% (2/12) 16.6% (2/12)	- - - - - - -		*p* = 0.64 *p* = 0.56 *p* = 0.56 *p* = 0.72 *p* = 0.55 *p* = 0.61 *p* = 0.59	
Medications - Statins - calcium channel blockers - oral antidiabetic drugs - angiotensin II receptor blockers/angiotensin converting enzyme blockers (ACE) - slow or/and intermediate acting insulin - immunosuppression therapy [corticosteroid, tacrolimus, mycophenolate mofetil (MMF)]	100.0% (13/13) 38.4% (5/13) 23.0% (3/13) 61.5% (8/13) 92.3% (12/13) 100.0% (13/13)	- - - - - -		100.0% (12/12) 50.0% (6/12) 25.0% (3/12) 58.3% (7/12) 91.6% (11/12) 100.0% (12/12)	- - - - - -		*p* = 0.53 *p* = 0.23 *p* = 0.51 *p* = 0.66 *p* = 0.47 *p* = 0.74	
Laboratory data								
Hematocrit (%)	44.2 ± 3.9	44.4 ± 4.2	*p* = 0.46	41.2 ± 3.8	41.0 ± 3.4	*p* = 0.65	*p* = 0.77	*p* = 0.06
Hemoglobin (g/dL)	15.3 ± 1.4	15.6 ± 1.0	*p* = 0.38	14.0 ± 1.2	13.9 ± 1.3	*p* = 0.89	*p* = 0.11	*p* = 0.24
Na^+^ (mg/dL)	139.8 ± 2.5	138.0 ± 2.6	*p* = 0.55	140.3 ± 4.3	140.3 ± 3.8	*p* = 0.92	*p* = 0.75	*p* = 0.38
K^+^ (mg/dL)	4.0 ± 0.2	4.0 ± 0.6	*p* = 0.66	4.3 ± 0.5	4.1 ± 0.4	*p* = 0.66	*p* = 0.15	*p* = 0.19
Ca^+2^ (mg/dL)	10.1 ± 0.5	10.0 ± 0.4	*p* = 0.87	9.7 ± 0.9	9.5 ± 0.6	*p* = 0.69	*p* = 0.94	*p* = 0.66
*p* (mg/dL)	3.4 ± 0.5	3.1 ± 0.4	*p* = 0.23	3.4 ± 0.4	3.3 ± 0.6	*p* = 0.72	*p* = 0.09	*p* = 0.01
M^+^ (mg/dL)	1.7 ± 0.2	1.6 ± 0.0	*p* = 0.62	1.6 ± 0.3	1.7 ± 0.2	*p* = 0.66	*p* = 0.50	*p* = 0.53
Fe^+^ (mg/dL)	90.4 ± 21.1	91.4 ± 19.8	*p* = 0.34	87.9 ± 16.6	88.1 ± 16.6	*p* = 0.45	*p* = 0.54	*p* = 0.18
Urea (mg/dL)	41.8 ± 8.8	37.0 ± 9.7	*p* = 0.02	40.6 ± 6.7	41.5 ± 6.4	*p* = 0.28	*p* = 0.83	*p* < 0.05
Creatinine (mg/dL)	1.2 ± 0.3	1.0 ± 0.1	*p* = 0.07	1.2 ± 0.5	1.2 ± 0.6	*p* = 0.91	*p* = 0.16	*p* = 0.36
Alkaline phosphatase (mg/dL)	70.8 ± 18.4	69.8 ± 20.1	*p* = 0.13	69.5 ± 9.7	69.9 ± 8.7	*p* = 0.47	*p* = 0.17	*p* < 0.05
Uric acid (mg/dL)	5.5 ± 1.2	5.3 ± 1.0	*p* = 0.14	5.8 ± 1.4	5.9 ± 1.1	*p* = 0.74	*p* = 0.23	*p* = 0.09
24-h urine albumin level (mg/dL)	111.4 ± 15.1	110.2 ± 14.8	*p* = 0.55	112.0 ± 18.7	112.9 ± 17.9	*p* = 0.62	*p* = 0.21	*p* = 0.01
FPG (mg/dL)	128.5 ± 27.3	101.0 ± 21.6	*p* = 0.02	126.6 ± 32.7	128.0 ± 31.1	*p* = 0.58	*p* = 0.49	*p* < 0.05
TC (mg/dL)	248.7 ± 29.7	239.0 ± 19.1	*p* = 0.08	250.9 ± 30.1	251.4 ± 29.7	*p* = 0.81	*p* = 0.56	*p* = 0.54
TG (mg/dL)	182.1 ± 13.9	151.2 ± 10.3	*p* < 0.05	178.0 ± 17.1	177.8 ± 18.2	*p* = 0.75	*p* = 0.18	*p* = 0.01
HDL (mg/dL)	48.3 ± 8.2	59.1 ± 7.6	*p* < 0.05	50.4 ± 6.4	49.9 ± 9.4	*p* = 0.28	*p* = 0.60	*p* = 0.11
LDL (mg/dL)	121.4 ± 10.9	120.8 ± 9.7	*p* = 0.09	119.9 ± 19.4	119.6 ± 18.9	*p* = 0.76	*p* = 0.68	*p* = 0.58
HbA1c (%)	6.8 ± 0.2	6.6 ± 0.4	*p* = 0.03	6.6 ± 1.0	6.6 ± 1.1	*p* = 0.95	*p* = 0.20	*p* = 0.21

Note: HD: Hemodialysis; BMI: Body mass index; KT: Kidney transplantation; eGFR: Estimated glomerular filtration rate; ESKD: End-stage kidney disease; CKD-EPI: Chronic kidney disease epidemiology collaboration equation; Na: Sodium; *p*: Potassium; Ca: Calcium; Mg: Magnesium; *p*: Phosphorus; Fe: Iron; FPG: Fasting plasma glucose; HDL: High-density lipoprotein; LDL: Low-density lipoprotein; TC: Total cholesterol; TG: Triglycerides; HbA1c: glycated hemoglobin. Data are expressed as mean ± SD; Significant at the 0.05 level (*p* < 0.05).

**Table 2:** Cardiorespiratory efficiency at baseline and the end of the study.

	Group A				Group B				A vs. B Group			
	Baseline	Follow-Up	*p*-Value	Intra-Observer Variability ICC (95% CI)	Baseline	Follow-Up	*p*-Value	Intra-Observer Variability ICC (95% CI)	Pre	Inter-Observer Variability ICC (95% CI)	Post	Inter-Observer Variability ICC (95% CI)
HRrest (bpm)	78.3 ± 15.0	75.4 ± 9.1	*p* < 0.05	0.8 (0.4/0.9)	77.9 ± 7.0	77.9 ± 7.7	*p* = 0.87	0.3 (−2.1/0.9)	*p* = 0.34	0.3 (−2.6/0.8)	*p* = 0.13	−0.5 (−0.5/0.8)
SBPrest (mmHg)	122.2 ± 12.3	118.6 ± 6.7	*p* = 0.23	0.4 (−1.5/0.8)	123.4 ± 9.5	123.3 ± 8.3	*p* = 0.86	0.2 (−3.2/0.8)	*p* = 0.26	−0.0 (−11.8/0.6)	*p* = 0.01	0.8 (0.2/0.9)
DBPrest (mmHg)	70.4 ± 4.7	68.6 ± 4.5	*p* = 0.16	0.4 (−1.2/0.7)	70.0 ± 4.0	69.9 ± 3.2	*p* = 0.78	−0.1 (−3.5/0.8)	*p* = 0.73	−0.1 (−4.8/0.7)	*p* = 0.23	0.4 (−1.5/0.8)
Time (min)	6.9 ± 1.1	7.5 ± 1.3	*p* < 0.05	0.8 (0.1/0.9)	6.8 ± 0.4	6.9 ± 0.3	*p* = 0.53	0.1 (−1.7/0.7)	*p* = 0.47	0.1 (−3.5/0.8)	*p* = 0.02	0.8 (0.5/0.9)
METs (%pred)	65.2 ± 8.4	69.3 ± 11.2	*p* = 0.03	0.8 (0.4/0.9)	64.9 ± 6.7	65.0 ± 5.1	*p* = 0.45	0.1 (−1.2/0.7)	*p* = 0.69	−0.6 (−13.0/0.6)	*p* = 0.04	0.9 (0.7/0.9)
VO_2_peak (mL/kg/min)	21.8 ± 4.1	23.5 ± 3.6	*p* = 0.01	0.9 (0.6/0.9)	22.3 ± 5.0	21.9 ± 4.5	*p* = 0.41	0.3 (−2.1/0.8)	*p* = 0.12	−1.2 (−18.9/0.5)	*p* < 0.05	0.9 (0.9/0.9)
VO_2_/HRmax	12.8 ± 3.7	13.4 ± 3.3	*p* = 0.11	0.4 (−0.3/0.7)	12.9 ± 1.9	12.8 ± 2.1	*p* = 0.87	0.2 (−1.8/0.7)	*p* = 0.71	0.2 (−3.3/0.8)	*p* = 0.48	−0.6 (−5.4/0.6)
VE/VO_2_max	38.6 ± 3.0	37.7 ± 2.9	*p* = 0.66	0.6 (−0.4/0.9)	37.7 ± 4.1	37.6 ± 4.4	*p* = 0.63	0.1 (−1.2/0.8)	*p* = 0.44	0.4 (−1.5/0.8)	*p* = 0.71	−0.7 (−6.6/0.5)
VE/VCO_2_max	34.0 ± 5.4	33.1 ± 4.9	*p* = 0.71	0.9 (−0.1/0.9)	32.9 ± 3.9	32.7 ± 4.2	*p* = 0.51	0.4 (−1.6/0.7)	*p* = 0.57	0.3 (−2.1/0.8)	*p* = 0.34	−0.2 (−2.5/0.8)
HRmax (bpm)	137.7 ± 22.2	146.5 ± 22.0	*p* < 0.05	0.8 (0.6/0.9)	136.8 ± 8.4	137.0 ± 8.4	*p* = 0.56	−0.5 (−11.5/0.6)	*p* = 0.18	0.4 (−1.4/0.8)	*p* < 0.05	0.9 (0.8/0.9)
SBPmax (mmHg)	160.4 ± 16.3	152.7 ± 15.0	*p* < 0.05	0.9 (0.8/0.9)	159.8 ± 12.9	159.9 ± 11.3	*p* = 0.90	0.1 (−3.6/0.7)	*p* = 0.49	−0.5 (−11.7/0.6)	*p* = 0.03	0.9 (0.7/0.9)
DBPmax (mmHg)	66.4 ± 5.0	65.4 ± 5.2	*p* = 0.58	0.7 (−0.04/0.9)	66.6 ± 3.9	66.6 ± 4.1	*p* = 0.95	0.1 (−3.7/0.8)	*p* = 0.87	0.1 (−3.6/0.8)	*p* = 0.11	0.5 (−0.5/0.8)

Note: Group A: Exercise group; Group B: Control group; ICC: Intraclass correlation coefficient; 95% CI: CI: 95% confidence interval (lower bound/upper bound); METs: Metabolic equivalents for physical activity; VO_2_peak: Maximum oxygen consumption; RER: Respiratory exchange ratio; VO_2_/HRmax: Ratio between VO_2_ and maximum heart rate; VE/VO_2_max: Ventilatory equivalents for oxygen; VE/VCO_2_max: Ventilatory equivalents for carbon dioxide; HR: Heart rate; SBP: Systolic blood pressure; DBP: Diastolic blood pressure; Data are expressed as mean ± SD; *p* < 0.05: baseline vs. 6 months follow-up; *p* < 0.05: group A vs.B.

**Table 3:** Dynamometry and functional capacity results at baseline and the end of the study.

	Group A				Group B				A vs. B Group			
	Baseline	Follow-Up	*p*-Value	Intra-Observer Variability ICC (95% CI)	Baseline	Follow-Up	*p*-Value	Intra-Observer Variability ICC (95% CI)	Pre	Inter-Observer Variability ICC (95% CI)	Post	Inter-Observer Variability ICC (95% CI)
30-s STS (number of repetitions)	12.7 ± 2.8	14.0 ± 4.2	*p* = 0.01	0.8 (0.4/0.9)	12.4 ± 2.5	12.5 ± 2.3	*p* = 0.71	−0.05 (−3.5/0.7)	*p* = 0.86	−0.4 (−4.4/0.6)	*p* < 0.05	0.9 (0.6/0.9)
Upper limb strength (watts)	90.6 ± 16.1	130.1 ± 17.8	*p* < 0.05	0.9 (0.6/0.9)	90.4 ± 14.6	89.0 ± 13.5	*p* = 0.78	0.1 (−0.1/0.6)	*p* = 0.62	0.7 (−0.1/0.9)	*p* < 0.05	0.8 (0.5/0.9)
Lower limb strength (watts)	144.8 ± 49.5	175.4 ± 56.1	*p* = 0.01	0.9 (0.4/0.9)	141.5 ± 31.0	140.7 ± 30.6	*p* = 0.46	−0.2 (−2.9/0.6)	*p* = 0.89	−0.0 (−5.3/0.7)	*p* = 0.02	0.8 (0.6/0.9)

Note: Group A: Exercise group; Group B: Control group; ICC: Intraclass correlation coefficient; 95% CI: CI: 95% confidence interval (lower bound/upper bound); 30-s STS: Sit-to-stand in 30 s. Data are expressed as mean ± SD; *p* < 0.05: baseline vs. 6-month follow-up; *p* < 0.05: group A vs. B.

**Table 4:** HRV and HRT analysis results at baseline and the end of the study.

Group A					Group B				A vs. B Group			
	Baseline	Follow-Up	*p*-Value	Intra-Observer Variability ICC (95% CI)	Baseline	Follow-Up	*p*-Value	Intra-Observer Variability ICC (95% CI)	Pre	Inter-Observer Variability ICC (95% CI)	Post	Inter-Observer Variability ICC (95% CI)
Mean HR (bpm)	71.3 ± 9.3	70.3 ± 8.4	*p* = 0.35	0.1 (−0.7/0.6)	71.1 ± 4.7	71.3 ± 3.3	*p* = 0.79	−0.2 (−0.6/0.4)	*p* = 0.81	0.3 (−2.1/0.8)	*p* = 0.52	−0.09 (−5.8/0.7)
TP (ms^2^)	2408.4 ± 1728.3	2549.5 ± 1827.3	*p* = 0.01	0.9 (0.8/0.9)	2315.9 ± 1644.2	2319.3 ± 1645.8	*p* = 0.54	0.3 (−2.7/0.8)	*p* = 0.19	0.3 (−2.3/0.8)	*p* < 0.05	0.8 (0.3/0.9)
Mean 24-RR intervals (ms)	896.1 ± 130.4	916.9 ± 117.5	*p* = 0.69	0.5 (−0.4/0.7)	894.0 ± 319.2	896.6 ± 316.6	*p* = 0.67	−0.1 (−2.4/0.3)	*p* = 0.63	−0.01 (−5.1/0.7)	*p* = 0.66	−0.01 (−5.2/0.7)
Time domain variables												
SDNN (ms)	98.6 ± 8.7	127.7 ± 10.7	*p* < 0.05	0.9 (0.8/0.9)	97.8 ± 8.1	98.0 ± 8.3	*p* = 0.66	−0.08 (−3.9/0.6)	*p* = 0.76	0.5 (−0.8/0.9)	*p* = 0.01	0.7 (0.1/0.9)
SDANN (ms)	105.5 ± 38.5	139.1 ± 20.1	*p* = 0.01	0.8 (0.3/0.9)	104.7 ± 39.6	104.6 ± 4	*p* = 0.67	0.4 (−0.2/0.7)	*p* = 0.68	0.6 (−0.4/0.9)	*p* < 0.05	0.8 (0.7/0.9)
rMSSD (ms)	38.7 ± 23.2	51.9 ± 28.0	*p* = 0.03	0.8 (0.5/0.9)	39.0 ± 24.4	39.3 ± 24.4	*p* = 0.55	−0.08 (−10.8/0.5)	*p* = 0.78	−0.1 (−4.8/0.6)	*p* = 0.03	0.8 (0.6/0.9)
pNN50 (%)	6.4 ± 4.8	8.0 ± 5.0	*p* = 0.08	0.6 (−0.2/0.9)	6.3 ± 5.3	6.2 ± 5.3	*p* = 0.71	0.1 (−3.4/0.7)	*p* = 0.66	0.2 (−4.6/0.8)	*p* = 0.04	0.8 (0.7/0.9)
Frequency domain variables												
VLF (ms^2^)	2844.5 ± 1753.5	2088.4 ± 1401.2	*p* = 0.01	0.7 (0.4/0.9)	2832.6 ± 1107.0	2833.0 ± 1126.6	*p* = 0.71	−0.06 (−12.5/0.5)	*p* = 0.88	−0.09 (−5.8/0.7)	*p* = 0.03	0.7 (0.5/0.9)
LF (ms^2^)	292.8 ± 213.7	254.1 ± 199.7	*p* < 0.05	0.8 (0.6/0.9)	292.5 ± 168.9	293.0 ± 168.0	*p* = 0.82	0.3 (−1.5/0.6)	*p* = 0.47	0.1 (−3.1/0.7)	*p* = 0.01	0.9 (0.8/0.9)
HF (ms^2^)	706.4 ± 446.2	852.3 ± 502.1	*p* = 0.02	0.8 (0.7/0.9)	701.1 ± 338.2	700.5 ± 340.6	*p* = 0.79	0.2 (−1.6/0.7)	*p* = 0.57	0.1 (−0.7/0.7)	*p* < 0.05	0.8 (0.6/0.9)
LF (n.u.)	25.3 ± 11.8	18.1 ± 347	*p* = 0.02	0.9 (0.8/0.9)	25.1 ± 7.9	24.9 ± 8.7	*p* = 0.88	0.01 (−5.7/0.4)	*p* = 0.92	0.02 (−4.8/0.7)	*p* = 0.04	−0.2 (−0.6/0.5)
HF (n.u.)	62.8 ± 7.0	93.6 ± 9.5	*p* < 0.05	0.9 (0.7/0.9)	62.7 ± 6.8	63.0 ± 7.0	*p* = 0.81	0.1 (−2.9/0.8)	*p* = 0.77	0.3 (−2.3/0.8)	*p* = 0.01	−0.1 (−2.4/0.3)
LF/HF	2.4 ± 0.7	1.9 ± 0.5	*p* < 0.05	0.9 (0.8/0.9)	2.5 ± 1.3	2.5 ± 0.9	*p* = 0.78	−0.2 (−2.3/0.6)	*p* = 0.82	−0.7 (−15.6/0.6)	*p* = 0.01	0.8 (0.7/0.9)
HRT												
TO (%)	0.0 ± 0.0	0.0 ± 0.0	*p* = 0.80	−0.001 (−1.9/0.7)	0.0 ± 0.0	0.0 ± 0.0	*p* = 0.95	−0.004 (−2.6/0.7)	*p* = 0.95	−0.003 (−2.3/0.7)	*p* = 0.94	−0.06 (−4.2/0.5)
TS (ms/RR)	6.3 ± 5.6	7.6 ± 6.4	*p* < 0.05	0.9 (0.7/0.9)	6.1 ± 4.3	6.2 ± 4.2	*p* = 0.71	−0.001 (−0.6/0.6)	*p* = 0.75	0.2 (−3.2/0.8)	*p* = 0.02	0.8 (0.7/0.9)
Mean 24-RR intervals (ms)	851.9 ± 146.4	852 ± 180.5	*p* = 0.84	−0.1 (−2.7/0.6)	852.7 ± 174.6	852.1 ± 174.0	*p* = 0.67	0.1 (−1.7/0.8)	*p* = 0.56	0.1 (−0.7/0.7)	*p* = 0.64	−0.3 (−1.6/0.7)

Note: Group A: Exercise group; Group B: Control group; ICC: Intraclass correlation coefficient; 95% CI: CI: 95% confidence interval (lower bound/upper bound); HRV: Heart rate variability; HRT: Heart rate turbulence; TP: Total; RR intervals: Time intervals between two successive heartbeats; SDNN: Standard deviation of RR intervals; SDANN: Standard Deviation of the 5 min Average NN intervals; rMSSD: Root mean square of successive differences between normal heartbeats; pNN50: The number of pairs of successive NN (R-R) intervals that differ by more than 50 ms; VLF: Very low frequency; LF: Low frequency; HF: High frequency; ΤO: Turbulence onset; TS: Turbulence slope; Data are expressed as mean ± SD; *p* < 0.05: Baseline vs. 6-month follow-up; *p* < 0.05: Group A vs. B.

**Table 5:** Multiple regression analysis with SDNN as an independent variable at the end of the study.

Model	B	β	*t*-Test	*p*
Participation to exercise	312.34		5.313	*p* < 0.05
Age	−1.072	−0.382	−1.696	*p* = 0.16
KT vintage	0.246	0.192	0.524	*p* = 0.63
HD vintage before KT	0.272	0.434	1.055	*p* = 0.36
VO_2_peak	−6.353	−0.932	−6.416	*p* < 0.05
30-s STS	−0.498	−0.066	−0.404	*p* = 0.70
Upper limb strength	−0.166	−0.114	−0.841	*p* = 0.44
Lower limb strength	0.348	0.750	3.332	*p* = 0.02
FPG	−0.343	−0.303	−0.844	*p* = 0.46
HbA1c	7.315	0.123	0.349	*p* = 0.75
R^2^ = 0.817				
F = 9.245				

Note: KT: Kidney Transplantation; HD: Hemodialysis; VO_2_peak: Maximum oxygen consumption; 30-s STS: Sit-to-stand in 30 s; FPG: Fasting plasma glucose; HbA1c: glycated hemoglobin.

**Table 6:** Multiple regression analysis with VO_2_peak as an independent variable at the end of the study.

Model	B	β	*t*-Test	*p*
Participation to exercise	51.138		4.764	*p* = 0.01
Age	−0.209	−0.507	−1.183	*p* = 0.32
KT vintage	−0.480	−0.133	−0.266	*p* = 0.80
HD vintage before KT	−0.239	−0.693	−1.045	*p* = 0.37
SDNN	−0.162	−1.103	−5.055	*p* = 0.01
rMSSD	−0.035	−0.286	−1.486	*p* = 0.23
LF	0.004	0.228	0.564	*p* = 0.61
HF	0.000	0.031	0.086	*p* = 0.93
Upper limb strength	−0.043	−0.202	−1.110	*p* = 0.34
Lower limb strength	0.066	0.975	2.926	*p* = 0.06
FPG	1.807	0.262	0.790	*p* = 0.44
HbA1c	1.162	0.360	1.167	*p* = 0.27
R^2^ = 0.904				
F = 15.430				

Note: KT: Kidney Transplantation; HD: Hemodialysis; SDNN: Standard deviation of RR intervals; rMSSD: Root mean square of successive differences between normal heartbeats; LF: Low frequency; HF: High frequency; FPG: Fasting plasma glucose; HbA1c: Glycated hemoglobin.

## Data Availability

The data presented in this study are available upon request from the corresponding author. The data are not publicly available due to ethical restrictions.
